# Exploring a Dance/Movement Program on Mental Health and Well-Being in Survivors of Intimate Partner Violence During a Pandemic

**DOI:** 10.3389/fpsyt.2022.887827

**Published:** 2022-05-26

**Authors:** Yasemin Özümerzifon, Allison Ross, Tessa Brinza, Gina Gibney, Carol Ewing Garber

**Affiliations:** ^1^Gina Gibney Dance, Inc., New York, NY, United States; ^2^Sanctuary for Families, Inc., New York, NY, United States; ^3^Department of Biobehavioral Sciences, Teachers College, Columbia University, New York, NY, United States

**Keywords:** intimate partner violence, dance, physical activity, trauma, domestic violence, stress, mental health, PTSD

## Abstract

**Aims:**

The aim of this study was to explore the feasibility and benefits of a 12-session dance/movement program for intimate partner violence survivors' mental health and PTSD symptoms during the COVID-19 Pandemic. The specific purposes were fourfold: (1) To determine the feasibility of delivering a virtual dance/movement workshop program; (2) to examine the effects of the program on symptoms of PTSD and psychological distress; (3) to determine whether heart rate variability improves; and (4) to describe the individual and shared experiences of a subgroup of participants of the program.

**Methods:**

Forty-five women ages 23–48 years were randomized to a 12-session virtual creative dance/movement program or a usual care control group, and completed questionnaires about PTSD and mental health symptoms, general health, physical activity, and underwent a brief measurement of heart rate variability. A subset of the intervention group participated in a semi-structured focus group.

**Results:**

The results of the study showed that the female survivors of intimate partner violence who participated in the virtual workshops felt better, and they experienced improved affect and reduced tension. They found new ways to express themselves, attune to their bodies, learn new self-care habits, and build community as they engaged in the workshops. Over the course of the study, the participants' symptoms of PTSD and psychological distress lessened. There were no changes in heart rate variability.

**Conclusions:**

This complex study was successfully completed during a global pandemic and resulted in improvements in some mental health symptoms and overall well-being. Given the importance of this work with intimate partner violence survivors, further work exploring dance/movement workshops for participants virtually and in-person is needed.

## Introduction

Intimate partner violence (IPV) is a prevalent public health issue. It is a pattern of abusive behavior by an intimate partner in a dating or family relationship, wherein the abuser exerts power and control over the victim or survivor. IPV can be mental, physical, economic, or sexual in nature. Incidents are rarely isolated and usually escalate in frequency and severity over time. Intimate partner violence can culminate in serious physical injury, suicide, or death (1–5). The World Health Organization estimates that one in three women have been subjected to intimate partner violence in their lifetime (5). According to data from the U.S., over one in five women have experienced severe physical violence by an intimate partner at some point in their lifetime, translating to nearly 29 million U.S. women (6, 7). In addition, more than 43 million women have suffered psychological abuse from an intimate partner (7). Globally, reports from China, the United Kingdom, the United States and other countries suggest the incidence of IPV has exponentially increased during the COVID-19 pandemic (8). A report by the State of New York indicates that calls to the state's IPV hotline increased 30 percent in April 2020 compared to the previous year, and calls increased by an astounding 18 percent from February to March 2020. New York State Police also reported that IPV incident calls were up by 15 percent in March 2020 compared to the previous year (9).

A considerable body of research has demonstrated that individuals who are abused by their romantic partners are at a substantially elevated risk for development of mental health disorders that include depression, low self-esteem, psychological distress, and Post Traumatic Stress Disorder (PTSD) (10). At the physiological level, neuroendocrine dysregulation may play a role in PTSD. Like other stressors, IPV activates the biological stress systems of the sympathetic-adrenomedullary (SMA) axis and the hypothalamic-pituitary-adrenal (HPA) hormonal axis (11, 12). Long-lasting activation of these systems results in chronically elevated hormones and other biological changes affecting structures in the brain, nervous system and organ systems in the periphery. Chronic hyperarousal and stress responses may lead to elevated blood pressure, attenuation of immune responses, increased inflammation, and also may result in impairments in cognitive function and psychological problems, such as PTSD (11, 12).

Physical activity is recommended in public health guidelines as it improves both mental and physical health (13, 14). Physical activity of many types can improve common co-morbid conditions associated with chronic stress, PTSD and mental health disorders, particularly symptoms of depression, anxiety, and sleep disorders (15–17). A recent meta-analysis of the effects of physical activity interventions on PTSD found that exercise reduced symptoms of PTSD and depression in people with PTSD (18). Several other studies have shown that participation in acute and chronic physical activity is inversely associated with hyperarousal symptoms, and results in improved PTSD symptoms, reduction in depression, enhanced sleep quality, as well as reducing fear ratings to unpredictable and predictable threats (18–20). Several biological mechanisms whereby physical activity may benefit people with PTSD have been identified. These include activation of the endocannabinoid system (an important neuromodulatory system that helps the central nervous system respond to insults), attenuation of the hyperactive state of the HPA and SMA axes, activation of the immune system, and reduction in inflammation, lending biological plausibility to the role of physical activity in improving symptoms of PTSD (19, 21). The benefits of physical activity, and the effective types and volumes remain to be fully understood, but the literature to date supports the promising role of physical activity as part of comprehensive treatment for PTSD.

The arts, in the form of creative arts therapy as well as arts in health interventions, also have been utilized for people who have experienced trauma. It is necessary to clearly define these two terms as the study's intervention can be described as a dance intervention; however, the literature up to this point largely reflects art and dance therapy. Although creative arts therapies and arts in health fields share certain commonalities, there are several key features that distinguish the two disciplines. Specifically, creative arts therapies utilize the arts to accomplish specific clinical goals with clients and arts in health interventions seek to cultivate enhanced overall well-being in participants through engagement with creativity and art for recreation and enjoyment (22). The benefits of the arts in regards to individual and communal health is explored in both of these fields. However, by focusing on the present moment rather than past trauma and by employing highly trained artists, not therapists, to deliver the intervention, this study's intervention is aligned with arts in health principles and standards of practice (23).

According to current literature, both arts-based therapies and arts-based interventions have been utilized for people who have experienced trauma, but there is not a body of research that specifically looks at the impact of dance interventions for survivors of IPV. There are a few common findings amongst studies of creative arts and dance therapies on the psychological and social impact for survivors of IPV. For instance, both creative arts and dance therapies have been shown to improve self-regulation and address symptoms of abuse and trauma (24–27). Creative arts and dance therapy also have been found to increase communication skills (25–27) and build community as well as reduce social isolation (25, 26, 28). Beyond these findings, dance therapy specifically has been reported to aid in re-accessing positive emotions (29–31), to increase body awareness and connection (30–32), to improve positive self-concept and self-esteem (29, 32), to discover and rebuild healthy boundaries (30–32), and to create a grown capacity for change by practice (29, 31). Although these findings showcase the potential benefits of utliizing arts-based therapies for IPV populations, there is a dearth of research on arts in health, and in particular dance, interventions.

An unpublished pilot study of an in-person, eight session Gibney *Move to Move Beyond*™ (*MTMB*™) dance/movement workshop program that was conducted by our research showed significant reductions in the Intrusive Feelings symptom cluster on the PTSD Checklist for DSM-IV in female IPV survivors who participated in the workshops, compared to those who received usual care alone. The content of the workshops was similar to the current study, but with fewer and in-person sessions, and childcare was provided.

The scientific evidence on the effects of creative movement, dance, and physical activity on mental health in IPV survivors is very limited; there are few available studies and these studies have substantial methodological limitations, including an absence of randomized controlled trials. Nonetheless, the results of these studies of physical activity, dance, and other creative arts suggest that IPV suvivors can benefit from physical activity and creative movement/dance programs. Therefore, the purpose of this randomized controlled trial studying a group dance/movement program was fourfold: (1) To determine the feasibility of delivering a virtual dance/movement workshop program; (2) to examine the effects of the program on symptoms of PTSD and psychological distress; (3) to determine whether a physiological measure of stress, heart rate variability, improves; and (4) to describe the individual and shared experiences of a subgroup of participants of the program.

## Materials and Methods

### Study Design and Overview

This study was a randomized control trial of a 12-session dance/movement program in survivors of IPV living in New York City. It was conceived as an in-person study, however, due to the COVID-19 pandemic, and the subsequent stay-at-home orders, it was modified and entirely conducted virtually. Participants were randomized to a dance/movement program or a usual care control condition and outcomes included measures of general and mental health as well as physical activity. Qualitative data were collected in a subsample of intervention group participants.

### Participants

Participants were recruited from Sanctuary for Families (SFF), a non-profit IPV service organization based in New York City. SFF is dedicated to the safety, healing and self-determination of victims of intimate partner violence and related forms of gender violence. It is one of New York State's largest providers of trauma-informed, holistic services for those who experience gender-based violence (GBV), which includes IPV survivors and their children. The organization provides comprehensive services for their clients and their children, which include, but are not limited to, providing clinical support, shelter, legal assistance, and economic empowerment programs. Individualized psychotherapy is available to SFF clients and may include cognitive processing therapy, child parent psychotherapy, or trauma focused behavioral therapy. Additional support programs for parents and children are also offered. SFF services are available to any victim of intimate partner or gender-based violence regardless of gender, sexual orientation, marital status, or immigration status. The clients of SFF are primarily low-income survivors of IPV, and of those, more than 90% are women of color, immigrants, and their vulnerable children.

When the COVID-19 pandemic hit New York City in March 2020 it created an unprecedented public health and economic crisis, and the resulting quarantine disproportionately impacted victims of IPV. Survivors working with SFF reported an array of urgent concerns triggered or exacerbated by the crisis. The list of these concerns includes job loss, food and housing insecurity, court closures preventing or delaying life-saving legal remedies like orders of protection, dangerous visitation situations, technological challenges around remote schooling and other childcare issues, abusive partners restricting reproductive and other healthcare access, and immigrant clients' fears of seeking emergency shelter, police or medical assistance.

Sanctuary for Families has been an essential service provider since the first day of the pandemic. SFF pivoted rapidly to continue providing nearly all of its holistic, life-saving services after stay-at-home orders went into effect March 2020, converting all but its shelter services to remote platforms within a matter of days (personal communication with Sanctuary for Families staff; unreferenced). From March to December 2020, they delivered services to adults and children that include offering mental health counseling sessions *via* telehealth, running clinical and legal helplines, offering crisis intervention, safety planning, and case management *via* phone and video calls. It is under these conditions that this research project was conducted while clients were still under the pandemic lockdown and restrictions during Fall 2020 through Summer 2021.

### Recruitment

Sanctuary for Families staff and social work interns performed a critical role in recruitment over the course of the study. Information regarding the study was presented to SFF caseworkers *via* email, and at staff meetings. Key staff members who have frequent client interaction, such as leaders of support groups and survivor programs like the Economic Empowerment Program, received individual communications in which they were requested to assist in identifying potential participants among their clients and to inform potential participants about the study. Once identified, participants were recruited by SFF social work interns and research staff *via* word of mouth and through distribution of an electronic flier.

Recruitment for participants was conducted from December 2020 to June 2021. Four cohorts of participants were recruited during this timeframe. Inclusion criteria for this study comprised of being 18 years or older and being a survivor of IPV. The study was open to all gender identities. The PARQ-x questionnaire (33) was used to screen for health-related exclusions, which included pregnancy or the presence of any underlying health condition that would make it unsafe to exercise. Interested people attended an orientation session, during which the study was explained in detail and questions were answered. Informed consent was obtained at the end of the orientation in accordance with the policies and procedures of the Teachers College, Columbia University Institutional Review Board.

### Confidentiality Procedures

Participant safety and confidentiality was the highest priority throughout the study due to the vulnerable study population; additional study standards and procedures were employed beyond those required by the U.S. Federal Policy for the Protection of Human Subjects at 45 CFR 46 (34). Furthermore, SFF's strict security measures for in-person and online services were enforced. SFF research study staff were the only study personnel who had access to individuals' complete names and contact information. Communication with the participants outside of the virtual workshops and data collection was carried out solely by the SFF staff who were in touch with participants *via* phone calls, texts and ZOOM (ZOOM, RRID:SCR_002175) version 5.9.1. The rest of the research team had access only to de-identified data.

All data collection and workshop sessions took place online *via* ZOOM. During these sessions, research staff was required to log on from a secure and private location. Participants were also asked to join from a non-public space wherein other individuals, except for their young children, could not see their screens or hear audio feedback from sessions. In order to protect identities, participants logged on to the platform with their first names, or a name of their choosing, followed by their study identification number. Participants were also able to turn on and off their camera as necessary.

Along with confidentiality procedures, an emergency protocol was developed for all participant interactions. The emergency protocol outlined a list of contacts, including numerous clinical staff members from SFF and local emergency numbers. Additionally, participants were encouraged by SFF staff to have a safety plan in place. Potential safety concerns were reviewed and addressed by the research team during weekly lab meetings.

### Intervention: Move to Move Beyond (MTMB™)

Founded by Gina Gibney in 1991, Gibney is a New York City-based performing arts and social action organization that taps into the vast potential of movement, creativity, and performance to effect social change and personal transformation. Gibney's *MTMB*™ workshop model was originally created in 1999 in partnership with SFF. Founder, Gina Gibney, and her company of dancers worked closely with SFF clients and staff, primarily Dr. Beth Silverman-Yam who is the former Clinical Director of the agency. *MTMB*™ workshop model was created by bringing together the strengths of dancers and social workers. Dancers through years of training focus on expressing themselves using their bodies and building community using movement, and social workers hold particular expertise in providing mental health support and recovery to survivors. The program was also piloted with and gathered input from those who experienced IPV.

In order to address the pattern of power and control in IPV, choice-making was decided to be the single most important component of the *MTMB*™ workshop's framework. Creating space for participants' choice-making and honoring those choices can be an antithesis of what one might have experienced in an abusive relationship. In addition, Gibney's dance-based intervention was theoretically based on the renowned trauma expert Judith L. Herman, M.D.'s work.

According to Herman (35), trauma creates disconnection from self and others; therefore, a major component of recovery can be achieved by creating connection to self and others: “The core experiences of psychological trauma are disempowerment and disconnection from others. Recovery therefore is based upon empowerment of the survivor and the creation of new connections. Recovery can take place only within the context of relationships; it cannot occur in isolation.” In addition, there are three stages of recovery: establishing safety, retelling the story of the traumatic event, and reconnecting with others. In the first stage, addressing the body plays an important role: “Establishing safety begins by focusing on control of the body and gradually moves outward toward control of the environment. Survivors often feel unsafe in their bodies.” The third stage of the recovery, reconnecting with others, addresses isolation that is commonly experienced in IPV: “Helplessness and isolation are the core experiences of psychological trauma. Empowerment and reconnection are the core experiences of recovery” (35). Gibney's *MTMB*™ program four-part model was built to support the first and third stages of recovery:

1. *Look inside/Reflection:* Movement and creativity foster reflection and self-awareness that can lead to greater self-confidence.
2. *Speak through Movement/Expression:* Movement and creativity encourage choice and decision-making. These activities help participants listen to their inner voices, express their feelings, choices and thoughts using their bodies.
3. *Work Together/Connection:* Moving and creating in a group is an enriching experience with valuable social and artistic rewards. Working together validates the experiences of every member of the group and helps to feel more connected to one another.
4. *Take Care*™*:* Sharing of stress reduction and self-care tools so that participants can easily practice activities on their own outside of the workshops.

The model's first component, *Look Inside*, and second component, *Speak through Movement*, aim to create connection to self, which can lead to empowerment, and support taking control of one's body. The third component of the model, *Work Together*, can be important for both the first stage of recovery as well as the third stage, which is reconnecting with others. Workshops were created to provide a non-judgmental environment for participants to connect with others, to break the isolation and to practice rebuilding relationships. A key distinction between this dance-based intervention and dance therapy is that the model does not address the second stage of the recovery process, which is retelling the story of the traumatic event. Therefore, the workshops never delve into the past or trauma. Instead, the workshops focus on the present and positive aspects, such as strengths of the survivors, things that spark joy and what participants are looking forward to in their everyday lives. *MTMB*™ workshops take place at IPV shelters where participants receive counseling that supports the second stage of recovery by retelling of the story of the traumatic. Hence, the *MTMB*™ model is complimentary to the IPV agency's work.

The *MTMB*™ workshops follow a set workshop structure and include activities that range from icebreakers to breathing exercises to creative movement generation based on a prompt. Based on the *MTMB*™ model and workshop structure, facilitators alongside Gibney staff created a 12-workshop curriculum for the study so that all four cohorts could experience the same workshop content. This curriculum was an adaptation of *MTMB*™'s in^−^person workshops to a digital format so that activities could be facilitated safely during the pandemic. The theme of the workshops varied, such as admiration, gratitude and embracing transformation. Gibney facilitators are all trained in trauma theory, trauma-informed practices, IPV foundations, de-escalation techniques, and emergency protocols.

### Intervention and Control Groups

Following informed consent, participants were randomized to either a dance/movement intervention group or a usual care control group by a simple 1:1 randomization allocation ratio using a random number generator. Participants in the intervention group were offered the Gibney *MTMB*™ workshop model twice a week for 6 weeks for a total of 12 sessions, in addition to continuing their usual care with SFF. Sessions occurred every Monday and Friday for 90-min and, if a session fell on a holiday, the program duration extended beyond 6 weeks. Each session took place virtually over ZOOM; and participants and research staff alike were asked to adhere to the confidentiality policy for digital sessions. Participants received a *Take Care Card*™ containing simple creativity, stretching, and mindfulness exercises that they could practice outside of the workshops. SFF research staff distributed each workshop's *Take Care Card*™ *via* email following workshops.

Sessions were split into a 60-min workshop and approximately 30-min data collection pre- and post-workshop. Two Gibney facilitators led the group sessions throughout the entirety of the study. Study participants in each cohort experienced both facilitation styles as the facilitators rotated leading workshops. In addition to the training of Gibney facilitators mentioned above, SFF clinical staff members were present at each session in order to provide hands-on support should a participant experience any emotional distress, or they were reachable *via* phone.

The usual care control group participants continued to receive standard care at SFF, which could include any combination of crisis counseling, legal services, economic empowerment programs, and housing assistance. Participants were also provided with health infographics, such as healthy sleep habit tips, how to prevent the spread of germs during COVID-19, and nutritious food suggestions. The health tips were sent twice weekly by SFF research staff *via* email in order to maintain contact with the participants and to reinforce study retention. Control group participants also were offered the opportunity to participate in an optional movement workshop following their completion of the study.

### Instruments

Data collection for each workshop session included attendance and chronicling participants' affect and collecting a heart rate reading. The Feeling Scale (36) was used to measure participants' affective valence response before and at the end of each session. Heart rate was measured using Polar H10 devices (Polar Electro USA, Bethpage, NY) during a relaxation exercise and captured in the Elite HRV app (Elite HRV, Inc, Asheville, NC) before the workshop session and measured continuously during and shortly after the workshop was completed.

Gibney workshop facilitators completed a post-workshop field log in order to note any deviation from the workshop curriculum and to highlight moments or points of concern. These were reviewed after each workshop by researchers for fidelity purposes as well as to address any potential concerns surrounding participant safety with the SFF clinical staff.

### Outcome Measures

Pre- and post-intervention surveys were self-administered electronically through the Research Electronic Data Capture (REDCap, RRID:SCR_003445) version V7.3.5 (37, 38).

The Posttraumatic Stress Disorder Checklist-DSM IV (PCL-5) (39) was used to ascertain symptoms of PTSD in accordance with the DSM-5 diagnostic criteria, and the Kessler K-6 Nonspecific Distress Scale (40) was utilized to survey participants for any symptoms suggestive of psychological distress. Additionally, the surveys contained a series of sociodemographic and health questions adapted from the Behavioral Risk Surveillance Survey System Questionnaire (41), the Godin Physical Activity Questionnaire (42) and a single item physical activity measure (43, 44).

Heart rate variability (HRV) measurements were taken at baseline and at the end of the 12 sessions. HRV is the beat-to-beat variation in the heart beat and reflects the underlying physiological interactions between the autonomic nervous system, the baroreceptors (blood pressure regulation), and the control of respiration. HRV was recorded during a progressive relaxation exercise for ≤7 min *via* a Polar H10 heart rate sensor (Polar Electro USA, Bethpage, NY), which connected with the participants' smart phones through the Elite HRV app (Elite HRV Inc, Asheville, NC). Raw data were exported from the Elite HRV app into Kubios software (Kubios Oy; Kuopio, Finland) for analysis using standard time domains and power spectral analysis methodology (45). Data were examined for aberrant QRS complexes (i.e., heart beats) and artifact, and a 5-min segment with stable heart rate was analyzed. Root mean square of the successive differences (RMSSD) of the R-to-R intervals on the electrocardiogram was calculated. The RMSSD reflects the variability from heart beat to heart beat activity of the autonomic nervous system, principally in the parasympathetic control of heart rate (46). The natural log of the RMSSD [ln(RMSSD)] was calculated to normalize the distribution of the RMSSD for ease of interpretation, yielding a scale of 0–100 (46). The standard deviation of the R-to-R intervals (SDNN), and the low (LF) and high frequency (HF) bands were also calculated. Baroreceptor activity during rest (vagal nerve activity) is represented by the LF band and the HF band and reflects parasympathetic activity and respiratory cycle variation effects (46). The ratio of LF to HF is thought to represent the balance between sympathetic and parasympathetic activity of the autonomic nervous system, although this is controversial (46).

### Focus Group

All intervention group participants were invited to join an optional 45–60-min virtual focus group interview in order to learn about their overall experiences in the *MTMB*™ program at the end of the intervention period. The group interviews were facilitated by two research team members using semi-structured interview methods (47). Interview questions were developed in advance and used consistently across the focus group interviews. The interview question set consisted of eight open-ended questions in order to obtain a breadth of responses and to avoid potential bias.

All focus group interviews were held virtually over ZOOM and were audio-recorded. Interviews were transcribed and further rectified against facilitator notes in order to collate verbal and nonverbal responses in each respective interview. All ZOOM video recordings were deleted following transcription of interviews in order to maintain participant confidentiality.

Qualitative data consisted of focus group interview transcripts and field log reports. Synthesis and analysis of the qualitative data was conducted utilizing the case study method, which has been employed within a substantial number of mixed-methods studies as a means to test theories presented by quantitative methods (48, 49). Two researchers reviewed the qualitative data independently and determined themes. The themes were then discussed to determine the common patterns that emerged for both reviewers until consensus was achieved.

### Statistical Analyses

Statistical analyses were conducted using SPSS version 28.0 (IBM, Inc, Armonk, New York). Descriptive statistics for baseline characteristics were calculated as means and standard deviations, medians and interquartile ranges, and frequency, depending on the level of measurement and the normality of the data distribution. A series of repeated measures ANOVAs were conducted using SPSS GLM, with group (intervention and control) as the between factor and time (pre- and post-test) as the within factor, each with two levels. Comparisons between the pre- and post- session affect (feeling scale) were made using a paired *t*-test. Alpha levels were set *a priori* at *P* ≤ 0.05 for all analyses. Effect sizes were calculated for all comparisons, with partial eta squared (η^2^) calculated for ANOVAs and Cohen's *d* for *t*-tests. For η^2^, values of 0.01, 0.06, and 0.14 were considered to be small, medium and large effect sizes, respectively. In regards to Cohen's *D*, effect sizes of 0.2, 0.5, and 0.8, respectively were interpreted as small, medium and large effect sizes.

## Results

Of the 66 individuals who expressed interest, 53 people attended the study orientation. 46 provided informed consent and 45 participants completed the study. Those who declined to participate in the study were due to scheduling or unknown reasons. The participants' sociodemographic characteristics at baseline are shown in Table 1. All of the participants identified as female and ranged in age from 23 to 48 years. Over three quarters of the participants identified as Black, Indigenous, and People of Color (BIPOC). Two thirds of the participants had completed some college or technical school or were college graduates. Thirty-five (77%) of the participants reported having one or more children 18 years old or under living with them, and 10 (22%) were living without any children.

**Table 1:** Sociodemographic characteristics of participants in a dance-movement program.

**Variable**	**Intervention (SD)** ***n* = 25**	**Control (SD)** ***n* = 20**	***P*-Value**
Age (years)	35 (6)	38 (8)	0.182
**Ethnicity/race**			
Latina (%)	45.6	32.6	0.152
Black (%)	32.6	23.9	0.546
Asian (%)	10.9	8.7	0.617
White (%)	4.3	4.3	0.590
Pacific Islander/Native American (%)	0	2.2%	0.435
**Education**			
Some high school (%)	6.5	6.5	1.000
High school grad (%)	8.7	13.0	0.203
Some college (%)	13.0	15.2	0.287
College graduate or above (%)	23.9	10.9	0.182
Number of children ≤18 years of age at home	1.2 (0.9)	1.5 (1.2)	0.440

*Values are means (standard deviations) or percentages.*

*SD, standard deviation. There are some missing data*.

Table 2 shows mental health characteristics at the start of the study. The scores on the K6+ Distress Scale ranged from 8 to 30 out of a possible range of 6–30. Scores of 19 or greater indicate high levels of non-specific psychological distress and likely signify the presence of serious mental illness (40). More than one-half of the participants had scores showing high levels of distress at baseline. The PCL-5 questionnaire assesses symptoms of PTSD and scores range from 0 to 80, and scores ≤31 suggest probable PTSD (Weathers et al. 50). Our participant's scores on the PCL-5 ranged from 2 to 73, and 45% had scores in the range suggestive of PTSD.

**Table 2:** Baseline mental health-related characteristics of participants in a dance-movement program.

**Variable**	**Intervention (SD)** ***n* = 25**	**Control (SD)** ***n* = 20**	***P*-Value**
Diagnosed depressive disease %	13.0	6.5	0.383
K6+ distress score	21 (6)	20 (5)	0.681
Mental distress elevated %	24	27	
Mental distress normal %	31	44	0.787
PCL-5 total score	33.8 (17.6)	34.3 (20.0)	0.647
PCL-5 re-experiencing cluster score	9.0 (5.2)	8.5 (6.0)	0.767
PCL-5 negative cognition and mood cluster score	10.6 (8.1)	12.1 (8.5)	0.589
PCL-5 avoidance cluster score	4.3 (2.3)	3.6 (3.3)	0.322
PCL-5 arousal cluster score	10.0 (5.1)	10.3 (6.4)	0.871
Probable PTSD %	28.9	24.4	
Unlikely PTSD%	26.7	20.0	0.540

*Values are means (standard deviations) or percentages.*

*SD, standard deviation. There are some missing data*.

The scores on the Leisure-Time Activity Scale shown in Table 3 were on average considerably higher than 24 (50), indicating that the participants were physically active, meeting the recommended target of accumulating at least 150 min of physical activity per week; only one participant in each of the groups did not meet this target. Differences in the number of hours per day spent in housework were observed, with the intervention group engaging in fewer hours per day of housework compared to the control group participants. Time spent sitting on a typical day was similar across the two intervention groups.

**Table 3:** Baseline physical activity of participants in a dance-movement program.

**Variable**	**Intervention (SD)** ***n* = 25**	**Control (SD)** ***n* = 20**	***P*-Value**
Leisure-Time Activity Scale	58.7 (23.8)	53.2 (25.7)	0.252
Days of 30 min moderate-vigorous physical activity (days/week)	2.5 (2.1)	3.9 (1.8)	0.025
Housework (h/day)	2.5 (2.2)	3.9 (1.8)	0.025
Sitting (h/day)	3.5 (3.2)	3.8 (2.1)	0.728

*Values are means (standard deviations) or percentages.*

*SD, standard deviation. There are some missing data*.

As shown in Table 4, 69 percent responded that their general health was good to excellent, while the remainder considered their health to be fair to poor. The most frequently reported diseases or cardiovascular disease risk factors were high blood pressure, high cholesterol, diabetes mellitus, asthma, depressive disease, or elevated Body Mass Index (BMI). The majority abstained from alcohol or drank moderately and did not smoke.

**Table 4:** Health-related characteristics of participants in a dance-movement program.

**Variable**	**Intervention (SD)** ***n* = 25**	**Control (SD)** ***n* = 20**	***P*-Value**
General health	2.7 (1.0)	3.1 (1.1)	0.170
BMI	27.7 (8.0)	33.0 (9.8)	0.053
**Alcohol use**			
None	39.1	42.2	
1–3 drinks/week	8.8	2.2	
4–7 drinks/week	4.4	0	0.135
High blood pressure %	8.9	6.7	
Gestational hypertension %	2.2	8.9	
Borderline hypertension %	2.2	0	0.42
High cholesterol %	15.6	4.4	0.26
Asthma %	0	6.7	0.075
Cancer %	2.2	0	0.565
Arthritis %	4.3	0	0.314
Diabetes %	2.2	2.2	0.686

*Values are means (standard deviations) or percentages.*

*SD, standard deviation. There are some missing data*.

### Adherence

Attendance at the 12-session movement/dance workshops ranged from 1 to 11 sessions, with a median attendance of five (interquartile range 5.5) sessions. Thirty-eight percent of participants attended fewer than five sessions, and 27% participated in eight or more sessions. One participant randomized to the intervention group was unable to attend any sessions, but she was included in the intervention group for all analyses (intention-to-treat).

### Symptoms of Psychological Distress

Table 5 shows the K6+ Scale and PCL-5 scores before and after the intervention. For the K-6+ Scale, there were no significant time effects (*F*_(1)_ = 1.69, *P* = 0.210, η^2^ = 0.038), or time by treatment interaction (*F*_(1)_ = 2.89, *P* = 0.09, η^2^ = 0.066). The PCL-5 total score and cluster subscale scores show a significant time effect (*F*_(1)_ = 15.52, *P* < 0.001, η^2^ = 0.275), showing that both group's PCL-5 total scores improved over the intervention period. There were no significant time by treatment effects (*F*_(1)_ = 1.093, *P* = 0.302, η^2^ = 0.026), meaning treatment and intervention groups experienced a similar decrease in PCL-5 scores. Similar findings were seen for PCL Cluster B (Re-experiencing) where there was significant time difference (*F*_(1)_ = 4.030, *P* = 0.051, η^2^ = 0.089) and no significant time by treatment interaction (*F*_(1)_ = 0.39, *P* = 0.531, η^2^ = 0.010). PCL Cluster C Avoidance scores showed no significant time (*F*_(1)_ = 2.134, *P* = 0.152, η^2^ = 0.049) or treatment by time effects (*F*_(1)_ = 0.332, *P* = 0.567, η^2^ = 0.008). PCL Cluster D Negative Cognition and Mood scores also showed significant time effects (*F*_(1)_ = 11.49, *P* = 0.002, η^2^ = 0.219) and time by treatment interactions approached significance (*F*_(1)_ = 3.26, *P* = 0.078, η^2^ = 0.074). PCL Cluster E Arousal Scores had a significant time effect (*F*_(1)_ = 11.95, *P* = 0.001, η^2^ = 0.226) but not a time by treatment interaction (*F*_(1)_ = 0.005, *P* = 0.946, η^2^ <0.001).

**Table 5:** Mental health symptoms before and after a dance/movement workshop intervention.

**Variable**	**Intervention (*n* = 25)**		**Control (*n* = 18)**	
	**Pre**	**Post**	**Pre**	**Post**
K6+ score	21.0 (5.9)	20.6 (6.6)	20.7 (5.3)	23.8 (5.8)
PCL-5 total score	33.8 (17.8)	28.8 (17.0)	32.0 (19.1)	23.39 (19.3)
PCL-5 cluster B (re-experiencing)	9.0 (5.2)	8.2 (5.4)	7.8 (5.7)	6.2 (6.0)
PCL-5 cluster C (avoidance)	4.3 (2.2)	3.6 (2.5)	3.3 (2.2)	3.0 (2.3)
PCL-5 cluster D (negative cognition and mood)	10.6 (6.1)	9.2 (6.7)	10.8 (8.1)	6.1 (6.8)
PCL-5 cluster E (Arousal)	10.0 (5.5)	7.9 (4.9)	10.1 (6.5)	8.1 (5.6)

*Values are means (standard deviations).*

*SD, standard deviation*.

### Affective Valence

Comparison of the Feeling Scale scores before (*M* = 2.7, SD = 1.0) and after (*M* = 3.8 + 2.4) the workshop sessions (*t*_(22)_ = −2.187, *P* = 0.04, *d* = 2.426, 95% CI [−0.881, −0.021]) showed that affective valence, the quality of an event or experience to be perceived as being pleasant or unpleasant (51), improved for the participants attending the movement/dance workshops.

### Heart Rate Variability

Heart rate variability variables before and after the intervention are found in Table 6. There were no significant group (*F*_(1)_ = 0.152, *P* = 0.700, η^2^ = 0.006) or treatment by group interaction (*F*_(1)_ = 0.100, *P* = 0.659, η^2^ = 0.007) effects for the root mean square of successive difference (RMSSD). When the natural log of RMSSD (ln(RMSSD)) is taken, the group (*F*_(1)_ = 0.098, *P* = 0.757, η^2^ = 0.004) and group by treatment (*F*_(1)_ = 1.361, *P* = 0.254, η^2^ = 0.048) effects remain insignificant. Similar results were observed for the ratio of low to high frequency [Group: (*F*_(1)_ = 2.829, *P* = 0.104, η^2^ = 0.095); Time × Group: (*F*_(1)_ = 0.94, *P* = 0.762, η^2^ = 0.003)]. These values fall within the range of normal values for these variables (52).

**Table 6:** Heart rate variability before and after a dance/movement workshop intervention.

**Variable**	**Intervention (*n* = 25)**		**Control (*n* = 18)**	
	**Pre test**	**Post test**	**Pre test**	**Post test**
RMSSD (ms)	36.7 (26.8)	31.8 (19.9)	55.6 (37.7)	55.9 (14.4)
Ln (RMSSD)	3.38 (0.71)	3.2 (0.72)	3.7 (0.87)	3.9 (0.38)
SDNN (ms)	64.0 (31.7)	61.2 (32.2)	74.5 (40.1)	68.9 (19.1)
Low frequency/high frequency ratio	4.8 (3.3)	3.7 (2.7)	3.6 (2.5)	2.0 (1.5)

*Values are means (standard deviations).*

*SD, standard deviation*.

### Physical Activity

There was a significant time effect (*F*_(1)_ = 17.89, *P* > 0.001, η^2^ = 0.390) for physical activity (Table 7) such that both the intervention and the control groups experienced a significant decrease in their physical activity participation score. There was no significant group by time interaction effect (*F*_(1)_ = 1.412, *P* = 0.245, η^2^ = 0.048). The number of days the participants reported engaging in moderate to vigorous physical activity of at least 30 min duration remained stable over time (*F*_(1)_ = 0.147, *P* = 0.704, η^2^ = 0.004) with an absence of a significant group by time effect (*F*_(1)_ = 0.311, *P* = 0.580, η^2^ = 0.008). Concurrently, time spent sitting increased significantly over time (*F*_(1)_ = 12.435, *P* = 0.001, η^2^ = 0.242) and there was no significant time by treatment effect (*F*_(1)_ = 0.058, *P* = 0.811, η^2^ = 0.001). Participants in both groups reported an increase in time spent doing housework over the time of the intervention (*F*_(1)_ = 107.25, *P* < 0.001, η^2^ = 0.733), but there was no significant time by treatment effect (*F*_(1)_ = 2.261, *P* = 0.141, η^2^ = 0.055).

**Table 7:** Physical activity before and after a dance/movement workshop intervention.

**Variable**	**Intervention (*n* = 25)**		**Control (*n* = 18)**	
	**Pre test**	**Post test**	**Pre test**	**Post test**
Leisure-time activity score	58.7 (23.8)	34.2 (22.9)	53.2 (25.7)	39.4 (28.1)
Days per week meeting physical activity targets	2.5 (2.2)	2.8 (1.8)	2.8 (1.8)	3.1 (2.0)
Time spent doing housework (h/day)	2.5 (2.2)	3.6 (2.7)	3.65 (1.7)	4.6 (4.8)
Time spent sitting (h/day)	3.4 (3.2)	7.5 (6.2)	4.0 (2.2)	8.7 (6.8)

*Values are means (standard deviations).*

*SD, standard deviation*.

### Qualitative Findings

Out of the 25 individuals in the intervention group, 16 volunteered to participate in the focus groups. Over the course of analysis, six themes emerged from the participants' responses to be included in this paper: the *MTMB*™ model provided connection to self and the body; expressing oneself through movement; community building; relaxation/stress relief; elicitation of positive emotions; and transfer of self-care habits that were learned during the workshop to everyday life.

#### Connection to Self and the Body

The first theme that emerged from the focus groups is that the workshops provided an opportunity for participants to connect with their bodies. Participants reported that they had not paid attention to their bodies prior to the workshops and that the workshops allowed them to connect with their bodies by providing time and space to focus on themselves. One participant described:

I found myself really connecting to the music and to giving myself that time to connect, in a way, to my body, which I, you know, had disconnected from for a long time. So, it was nice to connect to my body again.

The workshops also allowed space for participants to discover something new about their bodies: “So, I've been breathing shallow this whole time and didn't realize it. And, with the workshop, I connected to the fact that – just listen to your body.” This sense of exploration and discovery was further supported by the field log data. As a facilitator wrote, “One participant shared that doing these workshops brought awareness to how she is using her body and how tight one side feels in particular because it is her dominant side in carrying and lifting her child.”

#### Expression Through Movement

The second theme that emerged from the qualitative data analysis is the impact that expressing oneself through movement can have. Participants reported that they enjoyed being able to express themselves non-verbally as well as expressing their feelings using movement. One participant mentioned:

I really enjoyed the movement. But the part of that of… being able to put my own words and my own feelings into movement. I felt like that helped me to express myself a little bit better and to be able to say what I didn't really know how to say.

Participants also noted that gaining more confidence as well as enjoyment of creating movement were among the reasons why expressing oneself through movement was impactful, as one participant shared:

I think that being able to create your own movement and share it with others was, kind of, like, empowering. In a way, it gives you confidence to be able to share with others and express your feelings with others, especially people you don't know. So, to do that in this workshop has taught us a lot of confidence, I would say.

#### Community Building

The role that the *MTMB*™ program played in community building is the third theme that emerged from the qualitative data. Participants reported having a shared experience with others and feeling supported by other participants during the workshops, as well as connecting with the workshop facilitators and study staff. One participant shared:

I would say that the whole experience itself is, like, breathtaking because you taking a bunch of women from different walks of life and you're connecting us all by one specific tool, which is breathing. And we're using this breathing to identify different feelings that different people are feeling, which is, kind of, cool that you're taking one thing and, you know, like, unifying different things.

The facilitators also witnessed community building during the workshop. As one facilitator shared in the field log, “Many times the [ZOOM] chat had side conversations like participants commenting or affirming another in the chat.”

#### Relaxation and Stress Relief

The *MTMB*™ workshops also provided relaxation and stress relief based on the responses of the participants. Participants reported that even if they were feeling overwhelmed when they joined a workshop, they were able to let go of the stress over the course of the workshop. This was achieved through alleviating the tension in their bodies, as one participant described:

I'm more relaxed (shakes hands loosely in a wave like motion). I'm not as tense (holds hands in fists) as I was. I mean, I dealt with a lot of pressures. And I was, like, I knew my body was so tense (holds hands in fists again). I think I shared this in the first couple of sessions, like, my body's just so, like, (holds hands in fists and pulls them close to chest, shoulders curve inwards). And now, I feel a lot better.

Another participant referenced the importance of this type of tension release specifically for those who experienced IPV:

I think it can be so helpful to women in this situation to release some of the pent-up energy, pent-up stress, pent-up pain and anger. And, I felt like there were times where I was able to release some of that for myself.

This was also witnessed within the group, as one facilitator recalls in the field log what a participant shared during one of the sessions:

One participant shared that she initially did not [want to] do this study because she felt like she had so much on her plate already. She said she was so glad she did because she felt so much better and even after the first workshop, she noticed a difference and slept better.

#### Positive Emotions

According to analysis, the *MTMB*™ model elicited positive emotions, including experiencing joy and moving from shyness or nervousness to self-assured. As participants shared, specifically experiencing fun: “I mean, we were having fun with doing silly things. So, it was fun. Like, you can have fun, you can be silly with people, and no one's judging you. That felt really nice.” and feeling more confident were the most common positive emotions reported: “Initially, I was also very nervous about the people I'm meeting and sharing. Along the line, I became so confident that I wished every day is a Monday or Friday just to join the workshop.”

#### Self-Care Habits

Participants also reported by participating in *MTMB*™ workshops that they created new self-care habits. Many shared that they were integrating the skills learned from the model into their everyday routines. Here a participant described how she applied what she took away from the workshop in her daily life:

The stretches definitely were effective because we're in front of our computers most of the day and night. And, this actually, the exercises that we learned, that we were, like, going over. I would do them. I would do them all throughout the day when I feel tense. And, anyway, even outside if I'm on an appointment and waiting, I would do stretches and it really helped. It really helped to relax for the rest of the day and to take everything that would come to me with ease. You know? I just want to try to take a moment, stop, and breathe.

## Discussion

This study aimed to evaluate the feasibility and potential effectiveness of the Gibney *MTMB*™ dance/movement program designed for survivors of IPV. To our knowledge, this study is the first randomized controlled trial that looks at the impact of a dance intervention on survivors of IPV. The participants in this 6-week virtual randomized control trial were IPV survivors who were young to early middle-aged adult women and were representative of the sociodemographic of the clientele of SFF. The majority were well-educated and identified as BIPOC. Over three quarters of the participants had one or more children <18 years of age living with them.

At the start of the study, more than half of the study participants were experiencing high levels of non-specific psychological distress and symptoms of PTSD, suggesting the presence of significant mental illness and probable PTSD in many of the participants. Nearly two thirds considered their overall health to be fair to good and a third described their health as very good to excellent. Many participants had one or more physician-diagnosed physical or mental health conditions. Being an IPV survivor or having PTSD is associated with adverse cardiovascular risk profiles (53–55), and it appears that many of the participants have factors that can elevate risks of developing future cardiovascular disease. Mainly, the participants engaged in healthy behaviors; most were physically active, engaging in sufficient physical activity to meet recommended targets for health (13), reported no or moderate alcohol intake, and only one reported smoking.

The trial revealed improvements in some variables over the time of the intervention, and, for the most part, the responses were not significantly different across the intervention and control groups when comparing post-test to pre-test responses. For several variables, the analyses showed low moderate effect sizes for time and/or group by treatment interactions, suggesting that Gibney *MTMB*™ dance/movement program may have a differential effect on the intervention group compared with the control group. Visual inspection of the data showed that, for some variables, the intervention group improved more than the control group, but due to factors such as low adherence or high variability of the scores on the outcome measures, it was not possible to detect significant differences between the groups. Nonetheless, the study did show evidence that participation in the Gibney *MTMB*™ dance/movement program benefitted the study participants.

A key finding of the study was that symptoms of PTSD (i.e., PCL-5 score) declined significantly over the course of the intervention in a similar manner in both study groups, with a large effect size. When examining the PCL-5 cluster scores, all but Cluster C Avoidance scores improved significantly over time. There was a large effect size for time in the Cluster D Negative Cognition and Mood Scores and Cluster B Re-experiencing Scores. Cluster E Arousal Score also improved significantly over time, although with low to moderate effect sizes. The group by time interaction effects for the PCL-5 Total Scores or for the Cluster D Negative Cognition and Mood Scores, Cluster B Re-experiencing, and Cluster E Arousal did not reach significance. The Cluster D Negative Cognition and Mood Score approached significance with a moderate effect size. Cluster C Avoidance did not change over the course of the intervention nor were there differential effects across the two study groups. Although direct comparisons cannot be made with results obtained using previous versions of the PCL Checklist (56), it is notable that the results in an unpublished pilot study from our group found some similar findings with respect to PTSD symptoms. Using the PCL-4 in the pilot study, it was found that 75% of women in the movement/dance intervention group reported a significant decrease of an average of three points in PTSD related “Intrusive Thought” symptoms as compared to the 31% of women in the control group who reported a reduction.

The scores on the K6+ symptoms of psychological distress remained elevated in both groups for the duration of the intervention with no differential group effects and low to moderate effect sizes. In many participants, the scores on the K6+ continued to indicate substantial symptoms of distress at the study end. Though there were no significant differences detected, it is important to note that control group's K6+ scores increased over time as opposed to the intervention group whose scores remained roughly the same.

Heart rate variability was measured before and after the intervention in both intervention and control groups. None of the HRV variables showed any significant changes by group nor any significant interaction effects. There were many challenges to obtaining these measurements as a result of shifting the study to a virtual format. In order to gather these measures, a commercially available app, EliteHRV, was utilized. The app has been utilized successfully in other published studies (57), and has the advantage that the raw data is downloadable for analysis in the lab. Therefore, it was deemed as a reasonable alternative to collecting electrocardiographic (ECG) data directly as had been planned. While many recommend measuring HRV for durations of ≤24 h, this was not feasible given the nature of the living conditions of the participants. It was decided to use a shorter time period to sample the HRV as some authors have reported to be acceptable (57–59), although not all authors agree that short measurement times are valid (60). The measurement protocol for the HRV measurements involved the participants sitting comfortably (a supine position was not possible) and engaging in a progressive relaxation exercise during data acquisition. Unfortunately, many of the participants had young children present during data collection due to the closure of childcare centers during the COVID-19 pandemic. The participants were unable to place themselves in an environment free of distractions during the measurement periods as a result of this constraint. For this reason, the HRV data were obtained under less-than-optimal conditions, which undoubtedly contributed to the inability to detect any effects of the intervention on HRV.

In examining the results of the physical activity data, it is interesting to see that participants nearly doubled their daily sitting time by the end of the study, to an average level of 7.5–9 h per day. The increased sitting time could reflect changes in the participants' daily routine, such as starting a job training program or an internship; especially since some participated in the SFF Economic Empowerment Program that is designed to assist the clients in becoming financially independent. The participants also reported more time spent doing housework following the intervention, and this also may reflect that some participants moved out of a shelter into an apartment or other reasons that are not apparent.

The quantitative data as a whole provide encouraging, but not definitive, support for the benefits of the dance/movement workshops. For example, The PCL-5 Cluster D scores approached significance for differential responses with a moderate effect size, which suggests effectiveness of the program for Negative Cognition and Mood. Other measures of PTSD symptoms and psychological distress improved, although not significantly. Prominently, there was an acute positive effect of the workshops on affect. The participants uniformly reported more positive affective valence (feeling scale scores) at the end of the workshop sessions, compared to the start of the workshop, showing that the workshops helped them feel better.

Moreover, the qualitative data elucidated several positive effects of the program. Results of the focus group provided additional evidence that the participants found the program to be helpful, enjoyable and that the program had benefits in six areas: creating connection to self and the body; expressing oneself through movement; community building; relaxation/stress relief; eliciting positive emotions; and transfer of self-care habits that were learned during the workshop to everyday life. The results of the qualitative data confirmed that the implementation of theoretical components of the *MTMB*™ model: *Look Inside, Speak through Movement* and *Work Together* are effectual.

According to Herman's trauma and recovery theory, trauma creates disconnection from self and others, and therefore, an important component of recovery can be achieved by creating connection to self and others (35, 61). This theory corroborates that *MTMB*™ workshops may be able to support recovery for survivors from trauma by creating space for them to re-connect to their bodies. In particular, the first component of the *MTMB*™ model, *Look Inside/Reflection* is supportive of this connection. The qualitative findings are also consistent with previous literature that dance therapy increases body awareness and connection (30–32).

The qualitative data further intimates that participants discovered new, beneficial ways of expressing themselves through movement. This aligns with the second component of the *MTMB*™ model, *Speak through Movement/Expression*, where the aim is for participants to express their feelings, choices and thoughts using their bodies. Qualitative results supported that the *Speak through Movement* is impactful in expressing oneself non-verbally, as well as in providing an avenue to express emotions.

Herman's third stage of trauma recovery involves reconnecting with others (35). *MTMB*™ model's third component, *Work Together/Connection* is grounded in this principle and its cogency is bolstered by the qualitative data, which indicates that the aspect of community building was a positive impact noticed by participants across multiple cohorts. Therefore, the element of community building within the intervention might able to assist survivors' healing process in coordination with other mental health and social services. This finding also expands upon the evidence found in previous literature regarding art therapy's role in building community amongst survivors of IPV (25, 26, 28).

Another benefit of the program reported in the qualitative findings is relaxation and stress relief for participants. People who experience IPV might develop PTSD, which is associated with heightened stress, hypervigilance, and hyperarousal resulting from experiencing IPV (11). Therefore, interventions that include movement and aim to relax the body in order to aid in stress release may potentially support the recovery process from trauma by offering a complementary coping mechanism for stress and hyperarousal.

In addition to PTSD symptoms, IPV is associated with depression and re-experiencing intrusive symptoms (62). Therefore, the opportunity to have fun and to rebuild confidence can be a powerful experience that may allow participants to help break undesired patterns, such as intrusive thoughts, and show that finding joy after trauma is possible. Likewise, these qualitative results provide additional support to the quantitative finding that there was an acute positive outcome of the workshops on affect. This is also aligned with previous literature findings that dance therapy supports re-accessing positive emotions for survivors (29–31), as well as improves positive self-concept and self-esteem (32, 63).

The final finding from the qualitative data is that the program led to a transfer of self-care habits learned in the workshop to everyday life. This finding is worth consideration as it speaks to the program's potential to impact survivor well-being and lifestyle change beyond the intervention's end. It is vital that program participants are able to find time during their busy days for self-care given the many urgent issues they are potentially dealing with, such as food and housing insecurity and job loss. While additional follow-up is necessary to confirm this hypothesis, survivors reported utilizing tools and skills from the workshop in their daily lives at least in the short-term: in-between the workshops, as well as 1–4 weeks after the intervention has ended. As the title of the program suggests, *Move to Move Beyond*™ aims to help survivors along their journey beyond the confines of the workshop. Therefore, the reporting of the integration of self-care habits learned from the workshops into participants' daily routines is highly encouraging. Although further studies are needed to understand the long-term transference of these habits, in the short-term participants found time to practice the skills they learned in different contexts, including in their morning rituals and during time spent together with their children.

### Limitations of This Study

The effects of the COVID-19 pandemic on the research design, data collection, and the lives of the study participants cannot be underestimated. Due to delays in initiating the study as the result of all research being halted due to the global pandemic, it was necessary to compress the intervention to provide the 12 workshop sessions over approximately 6 weeks, rather than 12 weeks as originally planned. The research team believed that the increased frequency of sessions would allow completing the study cohorts in a timely manner, given the possibility that the pandemic may cause interruptions to the research. The twice-a-week format was also thought to provide a more powerful exposure of the intervention and the possibility of greater effect.

It is feasible that a more substantive exposure to the intervention (e.g., more sessions or longer program duration) or larger sample size might have been required to detect significant benefits in the psychological variables consequent to the intervention. The mild to moderate effect sizes observed for these variables hint that a larger sample size could be needed. The study sample size was estimated based on an 8-week pilot study of an in-person version of the Gibney *MTMB*™ workshop, and it is reasonable to conjecture that the responses to the virtual workshops and measurement procedures could have led to greater variability in the outcome measures, and thus more difficulty in detecting differences.

The need to shift the entire study to a virtual format was challenging for all. Many of the participants had smartphones as their only available device, some of which were aging. This situation frequently resulted in technical challenges with respect to Internet connectivity and, during measurement sessions, difficulties in toggling between ZOOM and the EliteHRV app. In addition, updates to the smartphone operating systems and the EliteHRV app sometimes created problems in connecting the Polar H10 devices to the smartphones *via* Bluetooth. The limitation of technology was also present during the workshops. Facilitators noted in their field logs that participants sometimes had unstable Internet, which at times affected the delivery of the curriculum: “We had some technical issues with one person having frozen video for a bit - so we didn't fully do dance circle.” Thus, technological challenges due to the Covid-19 pandemic not only played a role in how the data was collected for the study, but also how the intervention was administered throughout. This also emerged as a theme from the focus groups as one participant shared:

It was a little bit weird to be honest. You know, especially when you're doing dance, I feel like sometimes you feed off the energies of other people. And, while you know it's great to have ZOOM and to see people, it doesn't always come across through the screen. Especially because sometimes, I mean, and I understand and I respect it, but having the screen off makes it difficult. A little bit.

Before the pandemic, survivors attended the *MTMB*™ workshops without their children present in the room. During the period of time in which the study was conducted, all childcare centers that would normally be available to the participants were closed, requiring that those with children provide care for them within their place of residence. Some participants lived in shelters where they shared a single room with their children. In other cases, the women lived in apartments, but in New York City small living spaces do not always allow for easy detachment from others living in the home. Facilitators reflected this in their field logs as well: “[The participant] was very distracted through this session having to navigate crying kids and lots of movement through her space.” As a result, the presence of young children was a frequent distraction during the workshops and data collection. This emerged as a theme from our focus groups and qualitative analysis as one participant shared:

I liked most part of it. I mean, there was, um, just occasionally, um, I would be distracted. But not because of the workshop. But just, because, like, I had a baby around me. I had a toddler around me.

Furthermore, due to the group nature of the workshops and the necessity to collect data in small groups due to scheduling challenges, even the participants without children were exposed to the distractions the children may have presented. This was unavoidable given the circumstances, and the potential impact on participation in the intervention and confounding of the measurements, especially the HRV measurements cannot be discounted.

The attendance at the workshops was low, with half of the participants attending 5 or less of the 12 sessions provided. This resulted in lower exposure than expected, and the inability to detect significant changes in many of the outcome variables can undoubtedly be attributed to the low attendance. Participants were reminded of workshop sessions *via* text messages, the preferred mode of communication by the participants, on the day of the session, and SFF research staff followed up with anyone who missed a session. The workshops were intentionally scheduled to avoid Sanctuary for Family programming conflicts, but the participants may have had individual therapy, medical care, legal appointments, work, and other obligations that interfered with their attendance. Although specific programming was accounted for in arranging the workshop schedule, participants were simultaneously receiving services from SFF for which timing could not always be predicted.

The reasons for the erratic attendance are not fully understood, but some insight was provided by the focus groups. Participants experienced negative life changes due to the global pandemic. For instance, one participant reported loss of loved ones, “Even though the workshop was beneficial and it was good for me, you can't turn off the reality of life. In the midst of the workshop, I lost my brother and my father.” Interestingly, multiple participants reflected in the focus groups the desire to be present for workshops more frequently and expressed interest in continued workshops despite all the changes happening in their lives. The same participant that was grieving family members conveyed this feeling as well:

It's like, um, I look forward to more workshops because it seems as if before the workshop, I wasn't in the greatest space. But, during the workshop, it made me feel a lot better. I looked forward to joining and it bothered me on days that I had to miss.

Given the myriad of upheaval that survivors of IPV encounter on a daily basis, it is noteworthy that participants were able to join as frequently as they did throughout a global pandemic.

Difficulties notwithstanding, it is remarkable that this complex study was successfully completed in a population that is challenging to study due to their vulnerability. This is especially so because the study was conducted at a time in the participants' lives that is notoriously dangerous to their personal safety and fraught with high levels of stress and psychological distress (64, 65). The participants in this study had difficulties presented by the COVID-19 pandemic added on top of the typical adversities present in recovery from trauma (66, 67). In spite of the context of the individuals' lives and challenging circumstances at SFF with the rapid pivot in the nature of the delivery of services, there was a robust response to the recruitment efforts, which often were sent impersonally *via* email and not made through direct communications with the individuals. As a result of these efforts, the goal of 20 people completing the study was realized.

## Conclusion

This study showed that Gibney's *MTMB*™ dance/movement program helped the participants, who were female survivors of IPV, feel better. The participants reported experiencing enjoyment, improved affect, and reduced tension. They also found new ways to express themselves, attend to their bodies, learn new self-care habits, and build community as they engaged in the workshops. Over the course of the study, the participants' symptoms of PTSD and psychological distress lessened.

Future work is needed to expand the understanding of how a dance/movement program benefits IPV survivors. Studying optimal ways to provide this program virtually and in-person is needed. Though this study was open for recruitment to any gender identity, only women joined the study, therefore, looking at the impact of this work for male, transgender and gender non-conforming survivors of IPV is important. If including participants with children, providing childcare could allow more engagement in the workshops and more optimal conditions to collect outcome measures. Addressing technological barriers when delivering a virtual program is advisable, and providing devices such as a computer or tablet, reliable Wi-Fi, and more user-friendly measurement devices to all participants is recommended.

## Data Availability Statement

The datasets presented in this article are not readily available because, due to the vulnerable participants in this study, exceptional confidentiality procedures have been implemented to protect the identity of the participants. The data are restricted to ensure participant safety and cannot be shared. Requests to access the datasets should be directed to YÖ, yasemin@gibneydance.org.

## Ethics Statement

The studies involving human participants were reviewed and approved by Teachers College, Columbia University Institutional Review Board. The patients/participants provide their written and oral informed consent to participate in this study.

## Author Contributions

CG, YÖ, and AR were responsible for the design and conduct of the study, contributed to the literature search, and were responsible for the manuscript preparation. CG was responsible for the statistical analyses. YÖ was the project director. YÖ and TB were responsible for the qualitative data collection and analyses. TB was responsible for study coordination, data collection, and contributed to the literature search and manuscript preparation. GG was responsible for the intervention program design and contributed to the manuscript preparation. All authors contributed to the article and approved the submitted version.

## Funding

This project was supported in part by an award from *Research: Art Works* at the National Endowment for the Arts: Grant# 1856083-38-19 and the Laurie M. Tisch Illumination Fund. The National Endowment for the arts does not guarantee the accuracy or completeness of the information included in this paper and is not responsible for any consequence of its use.

## Author Disclaimer

The opinions expressed in this paper are those of the author(s) and do not represent the views of the Office of Research & Analysis or the National Endowment for the Arts.

## Conflict of Interest

GG, YÖ, and TB were employed by Gina Gibney Dance, Inc., AR was employed by Sanctuary for Families Inc. The remaining authors declare that the research was conducted in the absence of any commercial or financial relationships that could be construed as a potential conflict of interest.

## Publisher's Note

All claims expressed in this article are solely those of the authors and do not necessarily represent those of their affiliated organizations, or those of the publisher, the editors and the reviewers. Any product that may be evaluated in this article, or claim that may be made by its manufacturer, is not guaranteed or endorsed by the publisher.
